# Alkaline Phosphatase Protects Lipopolysaccharide-Induced Early Pregnancy Defects in Mice

**DOI:** 10.1371/journal.pone.0123243

**Published:** 2015-04-24

**Authors:** Wei Lei, Hua Ni, Jennifer Herington, Jeff Reese, Bibhash C. Paria

**Affiliations:** 1 Division of Neonatology, Department of Pediatrics, Vanderbilt University Medical Center, Nashville, Tennessee, United States of America; 2 College of Life Science, Northeast Agricultural University, Harbin, China; Konkuk University, REPUBLIC OF KOREA

## Abstract

Excessive cytokine inflammatory response due to chronic or superphysiological level of microbial infection during pregnancy leads to pregnancy complications such as early pregnancy defects/loss and preterm birth. Bacterial toxin lipopolysaccharide (LPS), long recognized as a potent proinflammatory mediator, has been identified as a risk factor for pregnancy complications. Alkaline phosphatase (AP) isozymes have been shown to detoxify LPS by dephosphorylation. In this study, we examined the role of alkaline phosphatase (AP) in mitigating LPS-induced early pregnancy complications in mice. We found that 1) the uterus prior to implantation and implantation sites following embryo implantation produce LPS recognition and dephosphorylation molecules TLR4 and tissue non-specific AP (TNAP) isozyme, respectively; 2) uterine TNAP isozyme dephosphorylates LPS at its sites of production; 3) while LPS administration following embryo implantation elicits proinflammatory cytokine mRNA levels at the embryo implantation sites (EISs) and causes early pregnancy loss, dephosphorylated LPS neither triggers proinflammatory cytokine mRNA levels at the EISs nor induces pregnancy complications; 4) AP isozyme supplementation to accelerate LPS detoxification attenuates LPS-induced pregnancy complications following embryo implantation. These findings suggest that a LPS dephosphorylation strategy using AP isozyme may have a unique therapeutic potential to mitigate LPS- or Gram-negative bacteria-induced pregnancy complications in at-risk women.

## Introduction

Clinical observations suggest that persistent or excessive inflammation from chronic, subclinical or inordinate infections of the female reproductive tissues or non-reproductive tissues are linked to an increased risk of developing general health problems and reproductive difficulties including infertility, spontaneous abortion, stillbirth and preterm birth (PTB) [1–5]. These clinical observations are supported by *in vivo* animal models that demonstrate pregnant mice infected with *E*. *coli* or injected (intraperitoneal or intraluminal) with lipopolysaccharide (LPS; the toxic membranous product of *E*. *coli*; usually known as endotoxin), face significant threat for early pregnancy complications such as: 1) poor embryonic development; 2) improper preparation of uterine horns for implantation; and 3) decidualization defects or embryo resorption following implantation [6–11].

Tissues recognize bacterial toxins by specific pattern recognition receptors, including Toll-like receptors (TLRs) [12]. Studies have revealed that TLR4 is the major LPS signaling receptor in mammals [13,14]. LPS recognition and responsiveness is strongly enhanced when LPS binds with cluster of differentiation 14 (CD14). Since CD14 has no ability to transduce a signal, it facilitates association of LPS with TLR4 [15–17]. TLR4 mediates LPS actions via cytosolic adaptor protein myeloid differentiation factor 88 (MyD88) or Toll-interleukin-1 receptor (TIR) domain-containing adaptor-inducing interferon-β (TRIF) [18]. Typical inflammatory responses of LPS via TLR4-MyD88 pathway include production proinflammatory molecules such as tumor necrosis factor (TNF)-α, interleukin (IL)-1β, IL6, and type II interferon (INF), IFN-γ. On the other hand, TLR4-TRIF signaling is primarily responsible for inducing production of type 1 interferons, IFNα and INFβ, and IFN-stimulated genes [19]. Previous studies have demonstrated that endometrial and decidual cells of women and mice express TLR4 and respond to LPS [20–22] and TLR4-MyD88 pathway promotes heightened inflammation in the uterus in response to LPS [23,24]. Despite these progresses in our understanding of the infection-induced inflammation response, maternal infection remains a major contributor of pregnancy complications and clinical therapies aimed at killing bacteria, suppressing inflammation or neutralizing the proinflammatory cytokine effects have proven ineffective in treating pregnant women with bacterial infection.

The body has several methods of detoxifying substances; one of those includes enzymatic conversion of substances from injurious to non-injurious. Detoxification treatments become necessary when the body’s natural detoxification system becomes overwhelmed [25]. The toxicity of LPS is associated with its Lipid A moiety consisting of two phosphorylated glucosamine units with attached six acyl chains (fatty acids) [26,27]. Studies have demonstrated that a potential mechanism of LPS detoxification involves dephosphorylation of Lipid A moiety by alkaline phosphatases (APs) [28,29]. In this context, studies have shown that a partially dephosphorylated Lipid A analogue, monophosphoryl Lipid A (MPLA) is virtually non-toxic compared with the intact Lipid A moiety [30,31]. Previous studies in non-gravid and gravid mouse uteri have revealed the AP activity patterns, but have failed to assign any specific physiological role of AP in the uterus [32–35]. Murine AP (EC.3.1.3.1) exists in several isoforms and is divided into two groups: tissue-nonspecific AP (TNAP) encoded by the *Alpl* gene and tissue-specific AP (TSAP) including duodenal intestinal AP (dIAP), embryonic AP (EAP) and global IAP (gIAP) encoded by *Akp3*, *Alppl2* and *Alpi*, respectively [36,37]. AP isozymes are endowed with dephosphorylating activity. A previous study revealed that the mouse uterus only expresses the *Alpl* gene [32]. If uterine AP isozyme contributes to LPS neutralization by removing a phosphate from the Lipid A moiety of LPS under physiological conditions, AP could be explored as a possible treatment strategy to prevent endotoxin-induced pregnancy complications.

In the present study, our first objectives were to specifically determine: 1) cells of which uterine compartment(s) have the ability to recognize LPS during the pre- and post-implantation period, and whether these uterine cells produce TNAP isozyme; 2) which TLR4 intracellular pathway is activated and responsible for the normal inflammatory process of embryo implantation, as well as LPS-induced uterine inflammation; and 3) does uterine AP isozyme dephosphorylate LPS? Earlier studies have shown that AP therapy has beneficial effects in endotoxin-induced sepsis in mice and in kidney injury in humans [38–40]. Despite this promising progress, no efforts have been made thus far to evaluate the efficacy of endotoxin detoxification as a means to prevent endotoxin-induced pregnancy complications. Therefore, we compared the *in vivo* effects of LPS and dephosphorylated LPS (MPLA) during early pregnancy in mice, and assessed the efficacy of accelerated LPS detoxification using one of the AP isozymes as a therapeutic intervention to protect early pregnancy from the harmful effects of LPS.

## Materials and Methods

### Animals and treatments

Adult virgin Crl:CD1 (ICR) females were purchased from Charles River Laboratory (Wilmington, MA) and housed under pathogen-free conditions on a 12 h light: 12 h dark cycle. All experimental protocols using these animals were approved by the Vanderbilt University Animal Care and Use Committee. Female mice were mated with fertile or vasectomized males of similar strain. Detection of vaginal plugs was designated as day 1 of pregnancy or pseudopregnancy [41]. Survival surgeries in mice were performed under general anesthetic using a mixture of ketamine (80 mg/kg) plus xylazine (10 mg/kg). Mice received Ketoprofen (5 mg/kg) for postoperative analgesic. All mice were euthanized by cervical dislocation under overdose of isoflurane. Ovariectomized mice were rested for 12 days prior to their use. All drugs used in this study were reconstituted in endotoxin-free water and injected using aseptic techniques to avoid contamination.

Whole uterine horns were collected between 0800 and 0900 h on days 1–4 of pregnancy. Embryo implantation sites (EISs) were collected at around 0900 h on day 5 after an injection (tail vein) of Chicago Blue B dye solution (0.1 ml of 1% dye in saline). EISs from days 6–8 of pregnancy were collected by visual identification at around 0900 h. To induce artificial deciduoma formation, sesame seed oil (20 μl) was injected into the luminal space of one uterine horn in day 4 pseudopregnant mice. Deciduomal tissues were collected 48 h after induction of decidualization [42].

To examine the early effects of LPS-induced TLR4 signaling in the whole uterus or at the EISs, LPS (100 μg in 0.1 ml/mouse) was injected intraperitonially (i.p.) into ovariectomized or day 5 pregnant mice. Purified LPS from *E*. *coli* serotype 055:B5 (Sigma, St. Louis, MO) was used based on evidence suggesting similar uterine endometrial inflammatory responses to different sources of LPS, including ultrapure LPS from *E*. *coli* serotype 0111:B4 (Invivogen) and purified LPS E. coli serotypes 055:B5 and 073:H16 [20]. The intraperitoneal injection of LPS was preferred based on previous studies demonstrating the presence of iodinated LPS in the placenta following intraperitoneal or intravenous injection of pregnant dams [43,44]. A subset of ovariectomized and day 5 pregnant mice also received an injection (i.p.) of either vehicle or MPLA (100μg/mouse, List Biological Laboratories, Inc., Campbell, CA). Drugs were not injected intraluminally in order to avoid uterine inflammation associated with surgical procedure, handling of the uterus with forceps and uterine distention due to fluid instillation. All mice were euthanized 6 h after drug injection. Whole uterine horns were collected from ovariectomized mice. Day 5 EISs were collected after blue dye injection. All tissues were flash frozen in cold Super Friendly Freeze’it and stored at -80°C.

Day 7 pregnant mice were treated with either vehicle or bovine intestinal AP (bIAP) by intravenous injection to determine whether systemic injection bIAP reaches at the EIS. Mice were euthanized 30 min after injections, and the liver and EISs were harvested. Frozen sections were stained for AP activity by histochemistry in the presence or absence of levamisole. Susceptibility to levamisole inhibition (TNAP inhibitor) was used to distinguish the presence of TNAP and bIAP in the liver and EISs.

To study the effects of LPS following embryo implantation, three doses (25, 50 or 100 μg/0.1 ml sterile saline/mouse) of LPS were injected (i.p.) on day 5 (0900 h). A subset of pregnant mice also received an injection (i.p.) of vehicle, MPLA (100 μg/mouse) or bIAP on day 5 of pregnancy. To investigate the protective role of AP against LPS, 150 units of AP from bovine intestinal mucosa (bIAP, Sigma) were injected into the mouse tail vein 5 min before the injection (i.p.) of LPS (100 μg/mouse). These mice later received an injection (i.p.) of bIAP (5 units) at 1500 h on day 5 and 0900 h on days 6 and 7 of pregnancy. All treated mice were euthanized around 0900 h on day 8. The number and wet weight of each EIS were recorded. Resorbed EISs were identified by their small size and a necrotic, hemorrhagic appearance when compared to normal EISs. Bovine IAP was used in our study because: 1) it is routinely used to dephosphorylate proteins [45] and nucleic acids [46]; 2) it has been used to prevent sepsis–induced mortality in mice [29,38] and kidney injury in humans [39,40]; and 3) commercially available bIAP has higher specificity (≥6,500 units/mg protein) than commercially available tissue-nonspecific AP (TNAP) (≥5 units/mg protein). Moreover, chimeric human recombinant TNAP (a.k.a. Asfotase alfa; Alexion pharmaceuticals, Lausanne, Switzerland) is not available given its pending approval by FDA (US) for clinical use in children with hypophosphatasia.

### RNA probe preparation

To generate partial cDNA clones, total RNA from day 1 uteri or day 6 EIS were subjected to RT-PCR using mouse antisense and sense primers listed in Table 1.

**Table 1:** Primers used for gene cloning and qRT-PCR.

Genes	Primer sequences (5’-3’)S, sense; AS, antisense	Accession#	Size(bp)	Application
*Tlr4*	S:ATTCAGAGCCGTTGGTGTATC; AS:TTCGAGGCTTTTCCATCCAATAGG	NM_021297	242	cDNA cloning
*Cd14*	S:CGCGGATTCCTAGTCGG; AS:CGCAGGAAAAGTTGAGCGAGT	NM_009841	267	cDNA cloning
*MyD88*	S:TCGAGTTTGTGCAGGAGATG; AS:ACTTGGTGCAAGGGTTGGT	NM_010851	333	cDNA cloning
*Trif*	S:CAGAAATTCTATAACTTTGTG; AS:TGGTTGATTTGATGCAGGCTCAGG	NM_174989	239	cDNA cloning
*Alpl*	S:GTGGATACACCCCCCGGGGC; AS:GGTCAAGGTTGGCCCCAATGCA	NM_007431	330	cDNA cloning
*MyD88*	S:ACTGAAGGAGCTGAAGTCGC; AS:AGTTCCGGCGTTTGTCCTAG	NM_010851	177	qPCR
*Trif*	S:TGAGGAGCCTCCAGACTTGT; AS:CCAGTCTGGTGTGTCAATGG	NM_174989	304	qPCR
*Alpl*	S:CCTGACTGACCCTTCGCTCT; AS:CTGCTTGGCCTTACCCTCAT	NM_007431	133	qPCR
*Tnfα*	S:GCCTCTTCTCATTCCTGCTTG; AS:CTGATGAGAGGGAGGCCATT	NM_013693	115	qPCR
*Il6*	S:AACGATGATGCACTTGCAGA; AS:GAGCATTGGAAATTGGGGTA	NM_031168	283	qPCR
*Ilβ*	S:CTGTGTCTTTCCCGTGGACC; AS:CAGCTCATATGGGTCCGACA	NM_008361	200	qPCR
*Rpl7*	S:TCAATGGAGTAAGCCCAAAG; AS:CAAGAGACCGAGCAATCAAG	NM_011291	246	qPCR

PCR-generated products were subcloned into pCR-II-TOPO cloning vector using a TOPO TA Cloning kit (Invitrogen, Carlsbad, CA). Nucleotide sequencing of these clones was performed to verify the identity and orientation of each clone. Plasmids bearing mouse cDNAs were extracted, purified, and linearized by the appropriate restriction enzymes. For *in situ* hybridization ^35^S-labeled antisense and sense cRNA probes were generated using appropriate RNA polymerases [47].

### *In situ* hybridization

*In situ* hybridization was performed as previously described [48]. Briefly, frozen uterine sections were fixed in cold 4% paraformaldehyde solution in PBS for 15 min on ice. Following prehybridization, sections were hybridized to ^35^S-labeled antisense probes at 45°C for 4 h. Sections hybridized with ^35^S-labeled sense probes were used as negative controls. After hybridization and washing, sections were incubated with RNase A at 37°C for 20 min. RNase A-resistant hybrids were detected by autoradiography using Kodak NTB-2 liquid emulsion (Eastman Kodak Co., Rochester, NY). The slides were counterstained with hematoxylin and eosin.

### Quantitative RT-PCR

Quantitative RT-PCR was performed as previously described [48]. Briefly, total RNAs from mouse uteri were isolated using TRIzol reagent (Life Technologies, Gaithersburg, MD). After DNA digestion by RQ1 deoxyribonuclease I (Promega, Madison, WI), total RNAs (2.5 μg) were reverse transcribed using oligo(dT) primers according to the manufacturer’s instruction (Invitrogen). The cDNA generated by RT was amplified using iQ SYBER Green Supermix kit (170–8880; Bio-Rad laboratories, Inc., Hercules, CA). The conditions used for qPCR were as follows: 95°C for 3 minutes followed by 40 cycles of 95°C for 10 seconds and 60°C for 30 seconds. All reactions were run in triplicate using the iQ 5 Real-Time PCR Detection System (Bio-Rad). Data from qPCR was normalized to ribosomal protein L7 (*Rpl7*), and analyzed using the 2^-ΔΔCt^ method. The primers used for qPCR are listed in Table 1.

### Histochemical detection of AP activity

Determination of AP activity by histochemistry was performed as described by Lei and coworkers [48]. Briefly, uterine cryosections (12 μm) were fixed in cold 4% paraformaldehyde solution in PBS and incubated with BCIP/NBT (5-bromo-4-chloro-3-indolyl phosphate/nitro blue tertazolium) substrate solution (Sigma-Aldrich, St. Louis, MO) for 1–2 min at 37°C. The histochemical reaction of AP was monitored under a stereomicroscope. Sections were rinsed in PBS and mounted using glycerol vinyl alcohol aqueous (GVA; Invitrogen) solution. As a negative control, some slides with mounted uterine sections were microwaved (highest power level) for 5–10 min in PBS prior to incubation with substrate solution [49]. To differentiate uterine AP isozyme forms, some slides were incubated with levamisole (TNAP inhibitor: 20 mg/ml) or L-phenylalanine (TSAP inhibitor: 20 mM) for 4 h at room temperature prior to addition of substrate solution. Sections were not counterstained in order to avoid obscuring phosphatase activity.

### Biochemical LPS dephosphorylation assay by uterine homogenates

A biochemical LPS dephosphorylation assay was carried out as described previously [48]. In brief, 2 μg protein extracted from day 1 uteri or day 7 EISs were added into a 100 μl Tris-HCl buffer (PH 7.6), containing 150 mM NaCl, 1 mM MgCl_2_ and 20 μM ZnCl_2_, with/without LPS, followed by incubation at 37°C for 3 h. As negative controls, heat-inactivated (95°C for 1 h) tissue lysates were added to the reaction mixture. To confirm the uterine AP isozyme involved in LPS dephosphorylation, uterine lysates were incubated with levamisole (5 mM) or L-phenylalanine (20 mM) on ice for 1 h prior to their addition to the reaction buffer. To detect inorganic phosphorus (Pi) released from LPS, malachite green solution (25 μl) was added for 10 min and activity was then determined from spectrophotometric absorbance readings (650 nm wavelength) taking into account the background readings. Each assay was performed in triplicate.

### Detection of LPS dephosphorylation at cellular sites of AP production and activity

Uterine sites of LPS dephosphorylation were identified histochemically as described previously [48]. In brief, cryostat cut uterine sections from early pregnant (days 1 to 8) uteri were fixed in formalin-Macrodex for 10 min and incubated with Tris/Maleic acid buffer (pH 7.6) containing MgSO_4_ and Pb(NO_3_)_2_ with or without LPS (3.2 mg/ml) for 2 h at room temperature. To ascertain whether uterine TNAP was involved in LPS dephosphorylation, sections were incubated with levamisole (20 mg/ml) at 37°C for 4 h prior to addition of reaction buffer with LPS. Slides were next washed with water, incubated with Na_2_S (2% in H_2_O) for 30 seconds to convert lead phosphate to lead sulphide, washed with water, post-stained with hematoxylin, dehydrated with ascending strengths of alcohol, washed in xylene and mounted with DPX mounting media (Sigma). Uterine sites of dark or light brown lead sulphide deposits were examined under bright field microscopy.

### Statistical analysis

Statistical analysis was performed using Student’s t test, or one-way analysis of variance (ANOVA) followed by Tukey’s test, with a p value <0.05 considered to be significant. Data are presented as means ± SD or SEM.

## Results

### Localization of *Cd14* and *Tlr4* mRNAs using *in situ* hybridization in the pre-implantation uterus and post-implantation EISs

**Fig 1:**
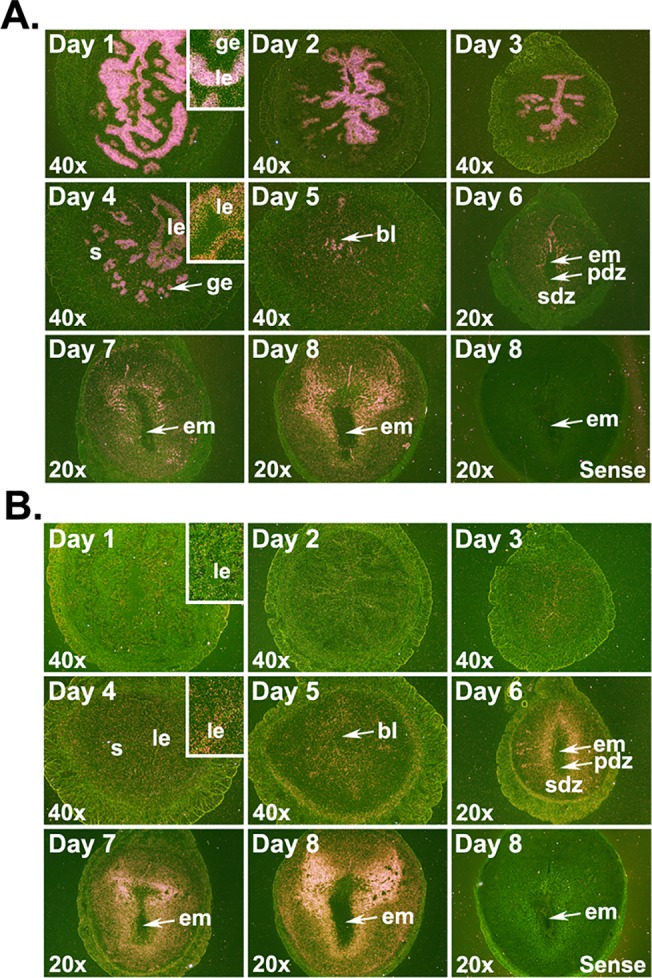
Localization of *Cd14* (A) and *Tlr4* (B) mRNAs in the early pregnant uterus. Uterine horns or implantation sites were collected from gestational days 1 to 8 for mRNA localization by *in situ* hybridization. Dark-field photographs were representative of three experiments. No hybridization signals were observed in sections hybridized with sense probes. Inserts show higher magnification (200X) of *Cd14* or *Tlr4* mRNA accumulations. bl, blastocyst; em, embryo; ge, glandular epithelium; le, luminal epithelium; pdz, primary decidual zone; s, stroma; sdz, secondary decidual zone.

We examined the localization of *Cd14* and *Tlr4* mRNAs to determine whether *Cd14* and *Tlr4* are expressed in similar uterine compartments. Prior to embryo implantation (days 1–4), *Cd14* mRNAs were mainly localized in the luminal and glandular epithelia with the strongest expression on day 1, and a gradual decrease from days 2 to 4 of pregnancy (Fig 1A). *Cd14* mRNA signals in the stromal compartment were detectable only on days 3 and 4 of pregnancy. Following implantation, signals were noted throughout the decidualizing stroma and in the mesometrial luminal epithelium (Fig 1A). While the uterine luminal and glandular epithelia showed low but detectable localization of *Tlr4* mRNA throughout the preimplantation period (days 1–4), the stromal compartment began showing *Tlr4* mRNA accumulation from day 3 and onwards (Fig 1B). Following blastocyst implantation, *Tlr4* mRNA accumulation in the decidua surrounding the implanted blastocyst progressively got stronger from days 5–8 and were noted in both the primary and secondary decidual zones (PDZ and SDZ, respectively; Fig 1B).

### *MyD88* and *Trif* mRNAs in the periimplantation uterus by qRT-PCR and *in situ* hybridization

**Fig 2:**
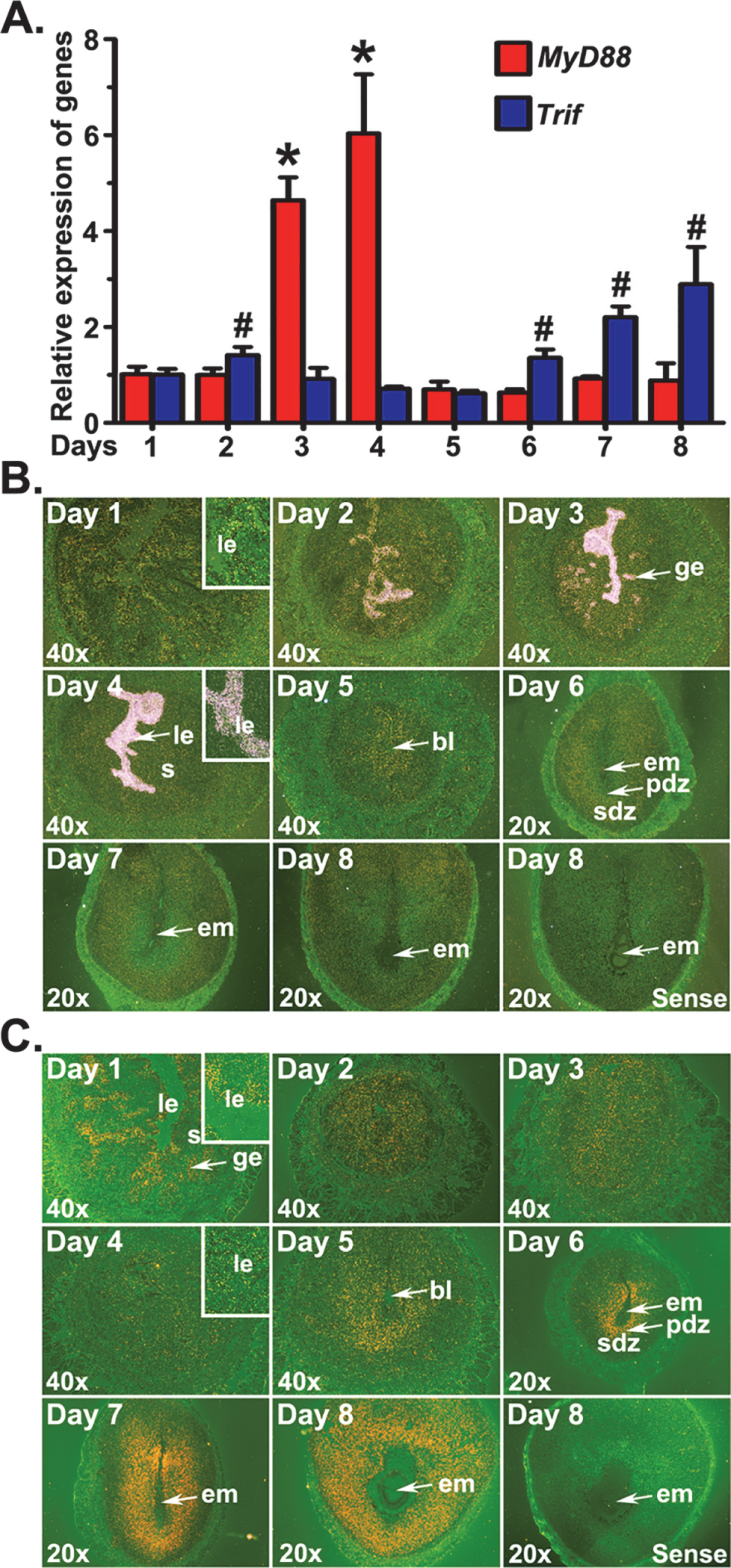
Expression of *MyD88* and *Trif* mRNAs in the early pregnant uterus. Uterine horns or implantation sites were collected from gestational days 1 to 8. (A) The levels of *MyD88* and *Trif* mRNA were determined by qRT-PCR, normalized to the housekeeping gene *Rpl7* and presented as the mean ± SD of three separate experiments (*,^#^p < 0.05; one-way ANOVA and Tukey’s test). Cell specific expression of *MyD88* (B) and *Trif* (C) mRNAs was determined by *in situ* hybridization. Dark-field photographs were representative of three experiments. No hybridization signals were observed in sections hybridized with sense probes. Inserts show higher magnification (200X) of *MyD88* or *Trif* mRNA accumulations. bl, blastocyst; em, embryo; ge, glandular epithelium; le, luminal epithelium; pdz, primary decidual zone; s, stroma; sdz, secondary decidual zone.

We analyzed the expression patterns *MyD88* and *Trif* mRNAs in the periimplantation uterus to demonstrate their association with uterine cellular events during early pregnancy. Results of qRT-PCR revealed that *Trif* mRNA expression levels were significantly greater in EISs from days 6 to 8 of pregnancy than preimplantation uteri, while *MyD88* mRNA expression levels were significantly greater in preimplantation uteri of days 3 and 4 (Fig 2A).

Localization of *MyD88 mRNAs* by *in situ* hybridization showed a restricted spatial and temporal expression pattern in the uterine epithelium during early pregnancy (Fig 2B). While a detectable signal was noticed in both the luminal and glandular epithelia on day 1, *MyD88* showed strong preferential expression in the luminal epithelium from days 2 to 4. The glands of the day 3 uterus also showed an abundant localization of the *MyD88* mRNAs. Following implantation, low accumulation of *MyD88* signals were noted in decidual cells surrounding the implanted embryo from days 5–8. *Trif* mRNA was specifically expressed in the luminal and glandular epithelia on day 1 of pregnancy (Fig 2C). However, the mRNA signals in the epithelium decreased from days 2 to 4. The uterine stromal compartment showed a detectable but gradual decrease in *Trif* mRNA signal from days 2 to 4 of pregnancy. Interestingly, strong accumulation of *Trif* mRNAs was noted in the decidual zone around the implanted embryos from days 5 to 8 of pregnancy.

### Uterine TNAP isozyme is involved in LPS detoxification during early pregnancy

#### 1) *Alpl* (TNAP encoding gene) mRNA levels and localization in the early pregnant uterus.

**Fig 3:**
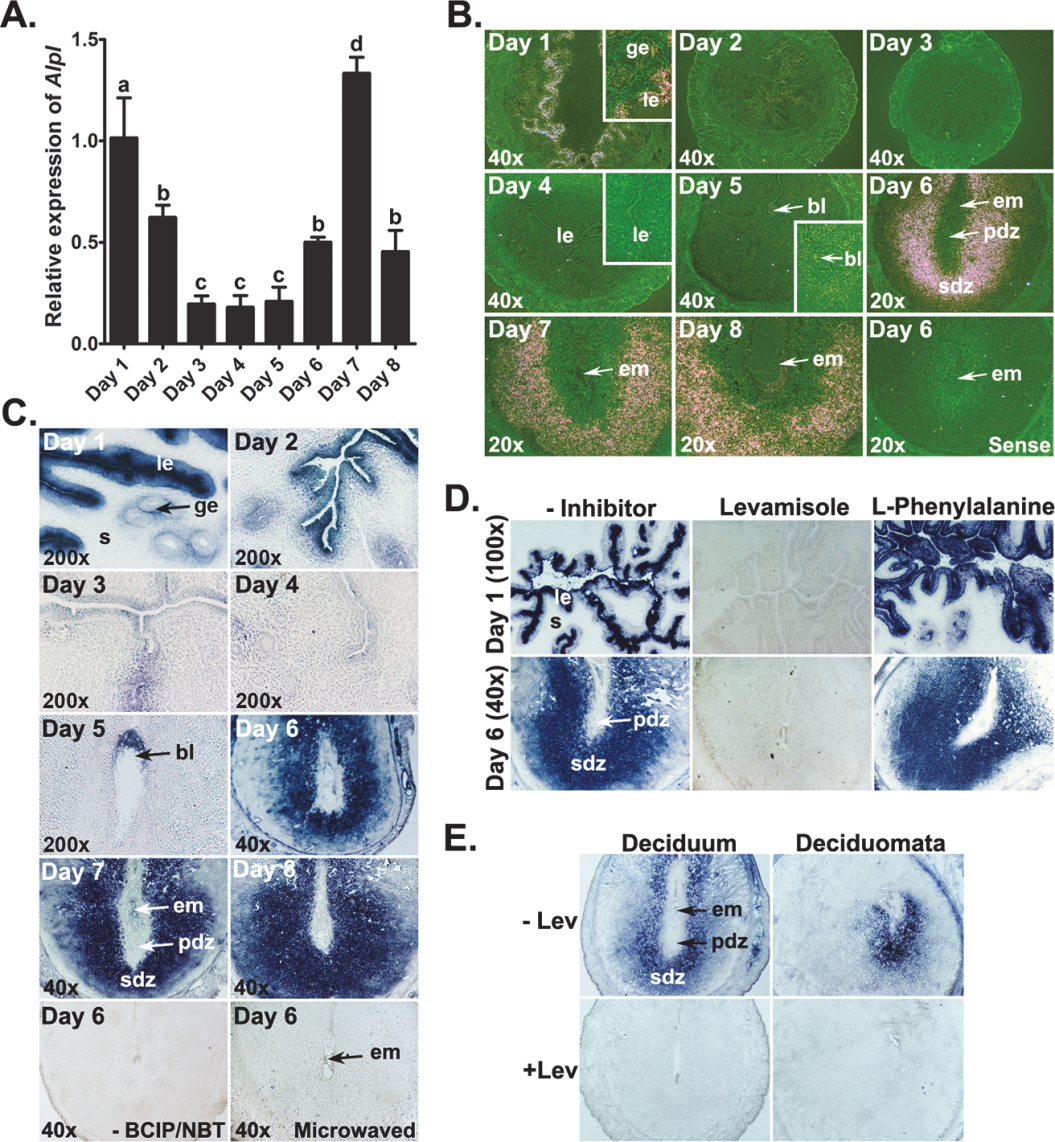
Expressions of *Alpl* mRNA and alkaline phosphatase (AP) activity in the early pregnant uterus. (A) The levels of *Alpl* mRNA were determined by qRT-PCR. Results were normalized to the housekeeping gene *Rpl7* and presented as the mean ± SD of three separate experiments, with different letters (a, b, c) indicating statistical difference (p < 0.05; one-way ANOVA and Tukey’s test). (B) Cell-type specific localization of *Alpl* mRNA was determined by *in situ* hybridization (n = 3). Inserts show higher magnification (200X) of *Alpl* mRNA accumulations. (C) AP activity patterns in the periimplantation uterus (n = 6). (D) Levamisole and L-phenylalanine were used as inhibitors of tissue-nonspecific AP (TNAP) and tissue-specific AP (TSAP), respectively. (E) TNAP activity localization in cells of the deciduum and deciduomata (n = 5). The embryo-induced decidua (deciduum) was collected on day 6 of pregnancy. The oil-induced decidua (deciduomata) was collected 48 h after intrauterine instillation of oil in day 4 pseudopregnant mouse. bl, blastocyst; em, embryo; ge, glandular epithelium; le, luminal epithelium; pdz, primary decidual zone; s, stroma; sdz, secondary decidual zone.

Using qRT-PCR (Fig 3A), we found declining levels of *Alpl* mRNA from days 1 to 4 of pregnancy. After embryo implantation, *Alpl* levels were significantly (p<0.05) higher in EISs of days 6–8 than EISs of day 5 of pregnancy. *Alpl* mRNA localizations were stronger in cells of the luminal epithelium (LE) compared to cells in the glandular epithelium on day 1. Reduced expression of *Alpl* was noticed in both epithelia on day 2 compared to day 1 of pregnancy with little or no signal observed in any uterine cell-types on days 3 and 4 of pregnancy. Following implantation, *Alpl* mRNA began to appear at low levels in the stroma surrounding the implantation chamber on day 5 of pregnancy (Fig 3B). As the implantation process progressed, SDZ zone surrounding the implanted embryo showed strong *Alpl* mRNA localization from days 6 to 8 of pregnancy. From day 5 and onwards, a faint *Alpl* signal was also noticed in cells of the implanted embryo.

#### 2) TNAP activity in the early pregnant uterus

The AP activity localization determined by histochemistry (Fig 3C) was similar to that of *Alpl* mRNA localization in the early pregnant uterus. AP activity was mainly located in cells of the luminal and glandular epithelia prior to implantation and the decidua following implantation (Fig 3C). AP activity in the luminal and glandular epithelia gradually declined from days 1 to 4. Although *Alpl* mRNA was not detected on days 3 and 4, minimal AP activity remained in the uterine epithelium. Epithelial AP activity almost disappeared on day 4 except for several isolated spots in the LE. AP activity was not observed in stromal cells of the preimplantation uterus. On day 5 of pregnancy, AP activity was found in stromal cells surrounding the implanted embryo, as well as the embryo. AP activity became more pronounced at the SDZ of the EISs from days 6 to 8 compared to day 5. Histochemical staining of day 6 EIS sections, in which AP substrate is omitted or enzyme activity is destroyed by microwaving, showed no positive color formation indicating the specificity of the histochemical method (Fig 3). Competition experiments in which the TNAP inhibitor (levamisole) [50] was added in uterine sections showed no positive AP staining in epithelial cells of day 1 uterus and cells within the decidua of day 6 EISs (Fig 3D) demonstrating the high specificity of TNAP activity. In contrast, sections incubated with the TSAP inhibitor L-phenylalanine [51] exhibited no inhibition of AP activity in day 1 uterus and day 6 EIS (Fig 3D). Histochemical staining revealed strong TNAP activity, which can be effectively blocked by levamisole, in cells of both the embryo-induced deciduum and artificial deciduomata (Fig 3E).

#### 3) LPS dephosphorylation by uterine TNAP

**Fig 4:**
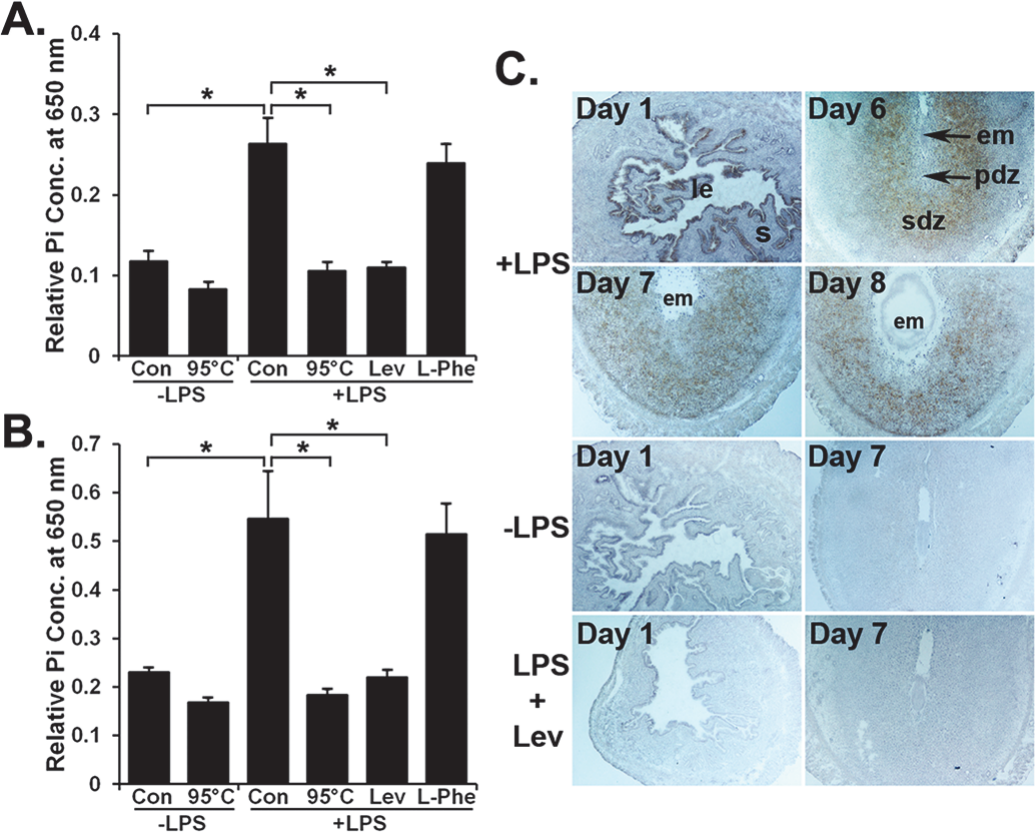
TNAP-specific LPS dephosphorylation in the uterus during early pregnancy. The uterine AP activity on LPS dephosphorylation was evaluated by biochemical assay in homogenates of the day 1 uterus (A) and day 7 embryo implantation sites (B). Enzyme activity was destructed while incubating homogenates at 95°C. Levamisole (Lev) and L-phenylalanine (L-Phe) were used as inhibitors of tissue-nonspecific AP and tissue-specific AP, respectively. Results are expressed as mean absorbance value ± SD of five individual experiments. Data were analyzed using one-way ANOVA followed by Turkey’s test (*p<0.05). (C) TNAP-specific LPS-dephosphorylation sites in uteri from gestational days 1 and 6–8. Bright-field images (40x) are representative of three experiments. No lead sulphide deposits were noted in sections from the day 1 uterus and day 6 embryo implantation sites in the absence of LPS or in the presence of LPS plus levamisole. em, embryo; le, luminal epithelium; pdz, primary decidual zone; s, stroma; sdz, secondary decidual zone.

Next, we evaluated whether uterine TNAP activity can cause LPS dephosphorylation and release of inorganic phosphorus (Pi). In this assay, uterine AP activity was measured biochemically using LPS as its substrate. Tissue homogenates from the day 1 uterus (Fig 4A) and day 7 EIS (Fig 4B) showed a significant release of Pi in the presence of LPS compared with controls in which LPS was omitted. Heat-inactivated tissue homogenates showed no significant effect on the basal Pi release. When levamisole was used in the assay to evaluate the contribution of uterine TNAP on Pi release from the LPS, we observed complete inhibition of Pi release from LPS by both tissue homogenates. In contrast, tissue homogenates-induced Pi release from LPS was not affected by addition of L-phenylalanine (Fig 4A and 4B).

Because we observed LPS dephosphorylation by pregnant uterine tissues in a biochemical assay, we considered that a histochemical study of LPS dephosphorylation in uterine sections would provide valuable information regarding possible sites of LPS neutralization in the uterus. Therefore, we investigated the pattern of LPS dephosphorylation in uteri from gestational days 1 and 6 to 8 (Fig 4C). On day 1 uterine sections, lead sulphide deposition was primarily found in cells of the luminal and glandular epithelium, but not in cells of the stroma (Fig 4C). Sections of EISs from days 6–8 showed strong lead sulphide deposits in the girdle of the secondary decidua, but not primary decidua. Control sections from day 1 uteri and day 7 EISs showed no lead sulphide deposits in any uterine cell types when incubated in buffer without LPS. Given that levamisole is a known inhibitor of TNAP activity, sections treated with levamisole together with LPS completely interrupted the phosphate release from LPS and showed no lead sulphide deposits in any uterine cell-types (Fig 4C).

### Assessment of inflammatory responses of LPS and MPLA in uteri of ovariectomized mice and in EISs of day 5 pregnant mice

#### 1) Inflammatory responses of LPS and MPLA in uteri of ovariectomized mice

**Fig 5:**
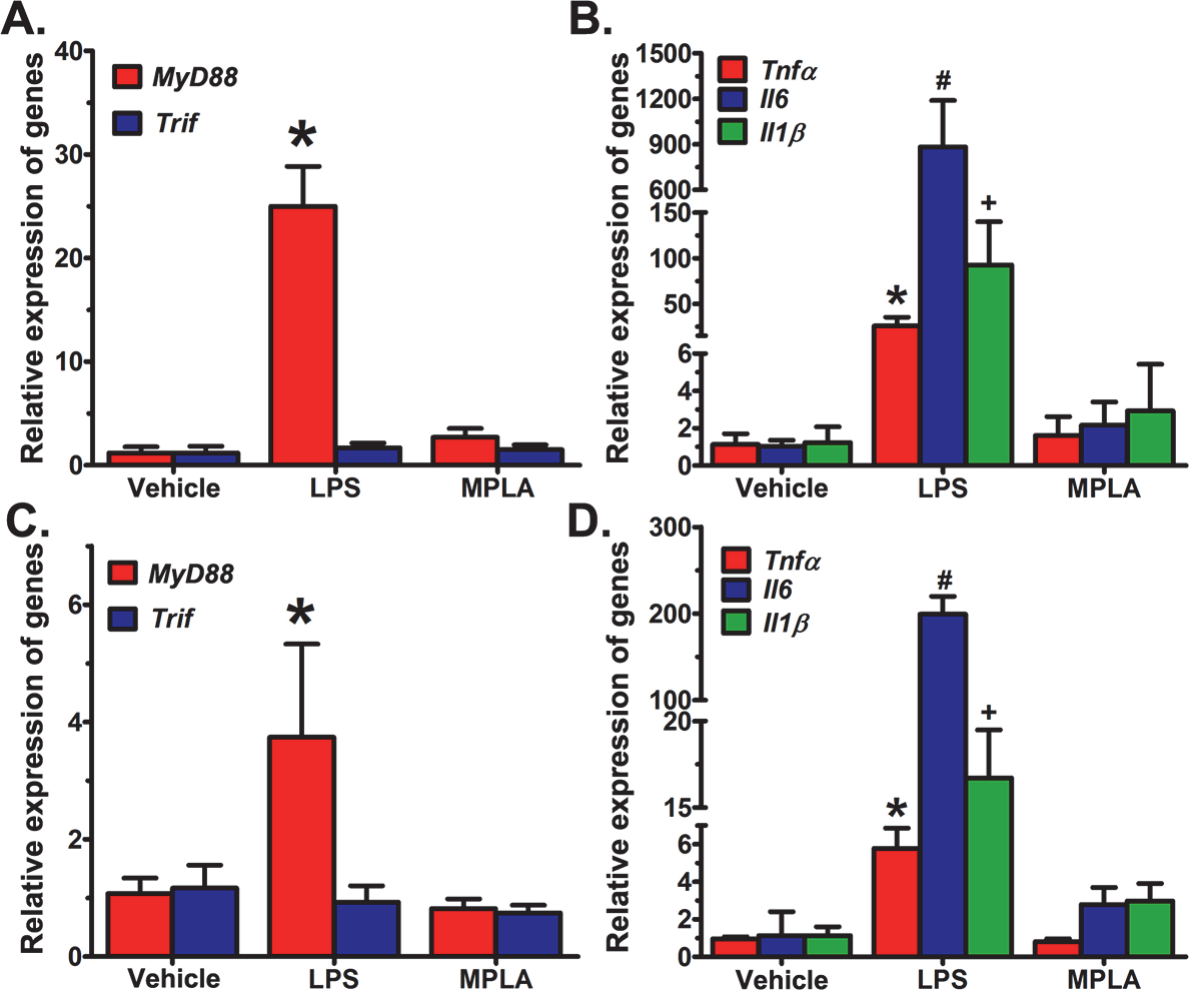
Effects of LPS on uterine *MyD88*, *Trif*, *Tnfα*, *Il6* and *Il1β* mRNA expressions. Relative expression of *MyD88* (*p < 0.05) and *Trif* (*p < 0.05) mRNAs in ovariectomized uteri (A) and day 5 EISs (C), respectively. Relative expression of *Tnfα* (*p < 0.05), *Il6* (^#^p < 0.05) and *Il1β* (^+^p < 0.05) mRNA in ovariectomized uteri (B) and day 5 EISs (D), respectively. All uteri were collected 6 h after vehicle, LPS (100 μg) or MPLA (100 μg) injection. Data were normalized to *Rpl7* and expressed as mean ± SD (n = 4), and one-way ANOVA followed by Tukey’s test, was used for statistical analysis.

To determine the influence of LPS and MPLA on expression of the *MyD88*, *Trif*, tumor necrosis factor-α (*Tnfα*), interleukin-6 (*Il6)* and interleukin-1β (*Il1β*) genes, ovariectomized mice were used to avoid the influence of ovarian hormones. Compared with the vehicle-treated group, *MyD88* mRNA expression showed a significant (p<0.05) increase in the LPS-treated group, but not in the MPLA-treated group. *Trif* mRNA expression showed no obvious induction in either LPS- or MPLA-treated mice (Fig 5A). Consistent with LPS-induced *MyD88* expression, LPS exposure in ovariectomized mice significantly (p<0.05) elevated the mRNA expression of *Tnfα*, *Il6* and *Il1β* (Fig 5B). In contrast, MPLA treatment was not effective in inducing *Tnfα*, *Il6* and *Il1β* mRNA expressions in the ovariectomized uterus compared with the vehicle treatment (Fig 5B).

#### 2) Inflammatory response of LPS and MPLA in the day 5 EIS

Compared with the vehicle-treated group, *MyD88* mRNA expression showed significant (p<0.001) increase in LPS-treated group, but not in the MPLA-treated group. *Trif* mRNA expression showed no obvious induction at the EIS in either LPS- or MPLA-treated mice (Fig 5C). Consistent with LPS-induced *MyD88* expression, LPS exposure significantly (p< 0.001) elevated the mRNA expression of *Tnfα*, *Il6* and *Il1β* at the EISs (Fig 5D). In contrast, MPLA treatment was not effective in inducing *Tnfα*, *Il6* and *Il1β* mRNA expressions at the EISs compared with the vehicle treatment.

### Histochemical detection of bIAP activity at the EIS following its injection

**Fig 6:**
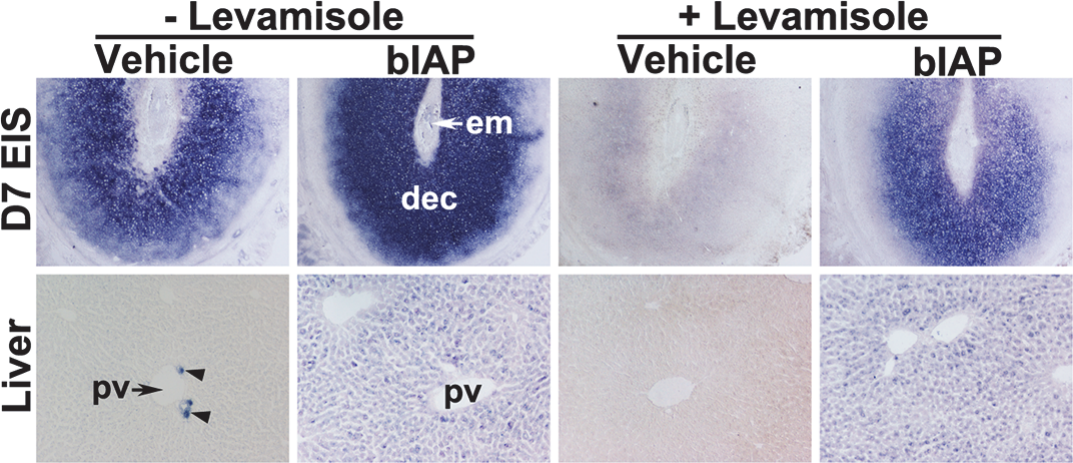
Accumulation of bIAP isozyme in the decidua following its systemic injection. Alkaline phosphatase (AP) histochemical staining in sections from the liver and embryo implantation site (EIS) from either vehicle or bIAP-treated day 7 pregnant mice. AP staining in the absence (left 4 panels) or presence (right 4 panels) of levamisole in the EIS (top, 40X) and liver (bottom, 100X) from the bIAP-treated mouse. pv = portal vein

Prior to exploring the therapeutic potential of AP in mitigating the LPS-induced pregnancy complications, we sought to identify whether systemic injection of bIAP is capable of reaching the EIS of pregnant mice. We demonstrated above that uterine TNAP activity is effectively inhibited by levamisole; consistent with previous reports that levamisole is an uncompetitive inhibitor of TNAP, but not TSAP [50]. This differential levamisole sensitivity between TNAP and TSAP (bIAP) served the basis for distinguishing endogenous uterine TNAP at the EIS and bIAP transported from the circulation to the EIS. We chose intravenous delivery of bIAP given the accessibility of the enzyme to its target tissues is largely dependent upon greater amounts of enzyme within the bloodstream [52]. After bIAP injection, we observed strong AP activity in the EIS and liver of bIAP-treated group relative to the vehicle-treated group (Fig 6). The liver of vehicle-treated mice showed AP activity in a very limited number of cells near the portal vein, but not in hepatocytes. However, hepatocytes of bIAP-treated mice showed positive stainings for AP activity. Compared to the decidua of EIS from the vehicle-treated mice, the decidua of the EIS from bIAP treated mice showed stronger AP activity. In addition, while AP activity at the EIS and liver from the vehicle-treated mice was completely inhibited by levamisole (Fig 6); AP activity at the EIS and liver from the bIAP-treated mice was not fully blocked by levamisole (Fig 6). These findings demonstrate the ability of circulating bIAP to reach the liver and EIS of the uterus within 30 minutes following injection.

### AP administration alleviated LPS-induced decidualization defects and resorption of implantation sites

**Fig 7:**
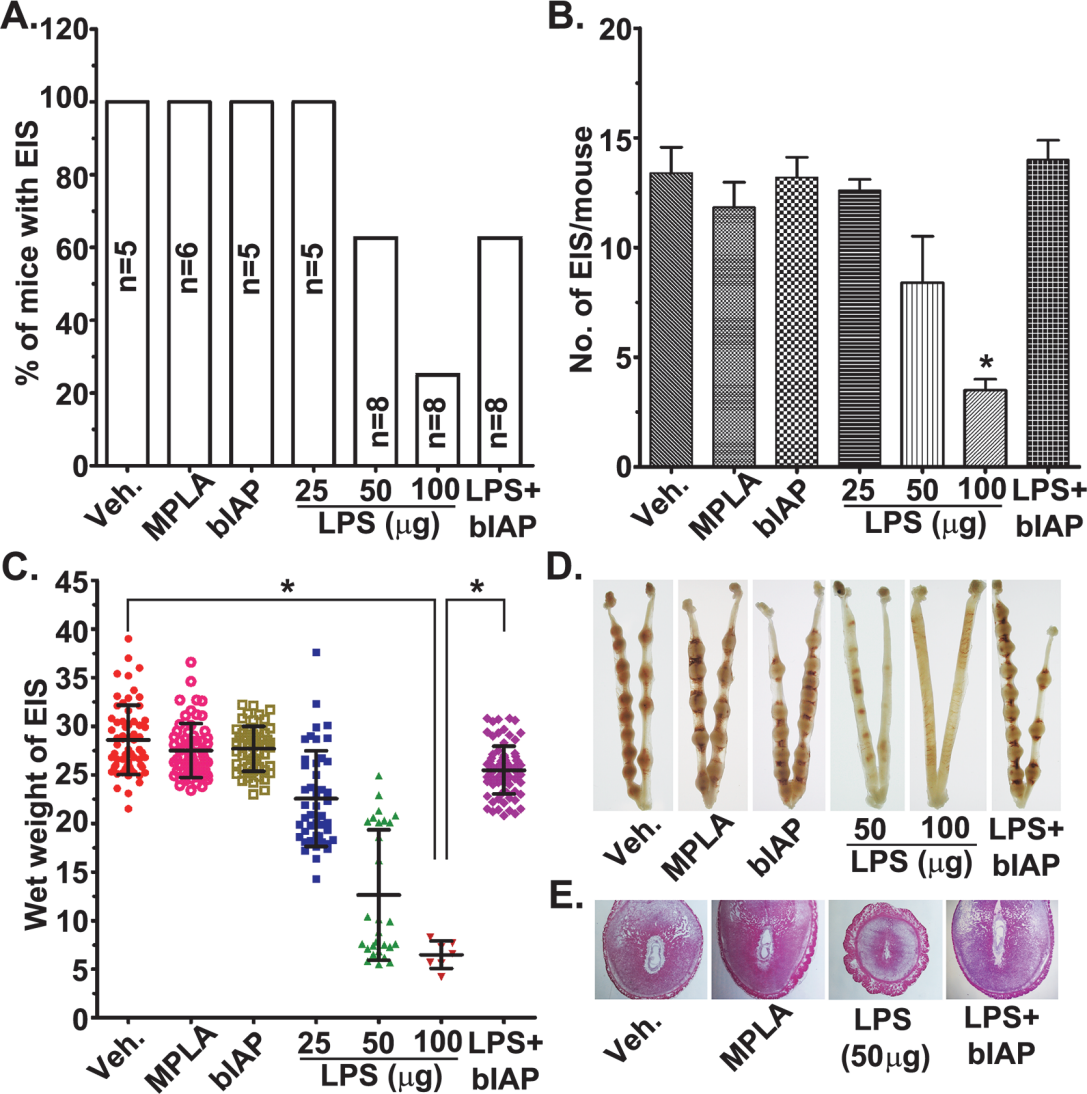
Alkaline phosphatase attenuates LPS-induced early pregnancy defects. Mice treated with vehicle, MPLA, bIAP, LPS or bIAP + LPS were analyzed for: % of mice with embryo implantation sites (EISs; A); Number of EISs (B); Wet weight of individual EIS (C). Morphological appearance (D) and histological analysis (E) of day 8 uteri from mice treated with vehicle, MPLA, bIAP, LPS or bIAP plus LPS. Bovine IAP (bIAP; 150 units) was administered intravenously (tail vein) 5 min before LPS (100 μg) injection. Mice in bIAP and bIAP + LPS groups received an additional injection of bIAP (i.p., 5 units) on day 5 (1800 h) and days 6 and 7 (0900 h) of pregnancy. * Results are presented as mean ± SEM. p<0.05 (ANOVA followed by Tukey test).

Pregnant mice treated with either vehicle-, MPLA- or bIAP on day 5 showed no effect on pregnancy outcome (Fig 7A and 7E) given the comparable number of normal looking EISs (Fig 7B and 7D) on day 8. Injection of the lowest dose (25 μg) of LPS revealed no decrease in the number of EISs, but showed a significant (p<0.05) decrease in the wet weight of the EISs compared to vehicle-, MPLA- and bIAP-treatment groups (Fig 7B and 7C). Mice receiving 50 and 100 μg of LPS exhibited significantly (p<0.05) fewer EISs as well as reduced wet weight compared to vehicle, MPLA or bIAP groups (Fig 7B and 7C). These adverse effects of LPS at the EISs were substantially (p<0.05) improved by the treatment of bIAP. While 25% of LPS-treated (100μg) mice had EISs, five out of 8 mice (62.5%) receiving LPS plus bIAP showed comparable EIS numbers, size and wet weight as observed in the mice treated with the vehicle (Fig 7A–7E).

## Discussion

Considering the importance of the uterus for pregnancy and fertility, the uterus likely has well designed mechanisms for recognizing and detoxifying bacterial endotoxin. In this study, we provide evidence that cells within uterine endometrium prior to implantation and the decidua following blastocyst implantation possess endotoxin recognition, cellular response and detoxification mechanisms necessary for immediate protection against endotoxin. An important observation of this study was that normal blastocyst implantation selectively stimulates the MyD88-independent pathway over the MyD88-dependent pathway at the EIS, while LPS exposure preferentially excites the TLR-MyD88-dependent pathway in the uterus and at the EIS. Additionally, we provide evidence that cells of the uterine epithelium and decidua are sites of immediate LPS detoxification by their endogenous AP isozyme and exogenous treatment of AP isozyme mitigates the inordinate LPS-induced pregnancy defects following implantation.

Studies have shown that TLR4 primarily mediates LPS responsiveness [53] since mice devoid of *Tlr4* are hyporesponsive to LPS [54]. In our study on the preimplantation mice uterus, *Tlr4* mRNA expression both in cells of the stromal and epithelial compartments of the uterus agreed with TLR4 protein expression reported previously [20,22] in the non-pregnant mouse uterus. Stronger *Cd14* mRNA expression in the uterine epithelial compartments compared with cells of the stromal compartment suggests that epithelial cells expressing strong CD14 are expected to have a higher affinity towards LPS and to be more efficient in LPS binding and signaling. Concomitant localization of *Cd14* and *Tlr4* mRNAs in the decidual compartment of the EIS following blastocyst implantation suggests that LPS binding sites are primarily located in cells within the decidua. These findings agree with those of Orgando and colleagues who showed CD14 abundance in endometrial glands and the decidua [55]. Taken together, our results suggests that cells of the uterine epithelium prior to initiation of blastocyst implantation and cells of the decidual compartment around the time and following blastocyst implantation are robust sites of LPS sensing.

Intracellular events following the formation of the LPS-CD14-TLR4 complex depend on recruitment of the adaptor protein MyD88 or TRIF. This was demonstrated by showing complete loss of nuclear factor kappa B activation in response to TLR4 stimulation in mice devoid of both MyD88 and Trif [18]. *MyD88* and *Trif* mRNA levels and localization in the uterus from days 1–8 of pregnancy showed event- and cell-type specificity. Our finding that elevated levels and strong accumulation of *MyD88* mRNAs occurs in cells of the uterine LE on days 3 and 4 of pregnancy supports the prior microarray data of Pan and colleagues [56] and suggest a connection with the transition of LE to the receptive state. However, mice devoid of *MyD88* are viable and fertile [24]. Thus, its requisite in inducing uterine receptivity is dubious. However, *MyD88*-deficient mice, drosophila, and humans have crippled defenses against a plethora of pathogens [57–59]. Given this crucial defensive role of MyD88 against pathogens, it is not surprising that this molecule showed strong expression during the uterine receptive phase that allows blastocyst implantation. It is therefore conceivable that uterine cell-type specific utilization of MyD88-dependant pathway during preparation for implantation may lead to the production of precise amounts of proinflammatory mediators, which may contribute to innate protective response. In contrast to *MyD88* expression patterns during early pregnancy, we observed unexpected upregulation of *Trif* mRNA expression in cells within the decidua as a result of blastocyst implantation. Stromal cell decidualization at the EIS is considered a local proinflammatory event [60]. Moreover, the decidua is also a place of invading, proliferating, dying and repairing cells. Thus, the dominance of *Tlr4* and *Trif* in the decidua in response to implantation is perhaps a reflection of explicit molecular inflammatory events within the decidua to protect both the mother and offspring. This prediction is supported by the observation that interferon stimulated gene 15 (a target gene of TLR4-TRIF signaling) expression is only found in decidual cells, but not deciduomal cells [61]. Together, these results suggest the uterine cell- and event-specific modulation of MyD88 and TRIF signaling during the periimplantation period, but how this TLR4 signaling paradigm shift relates to harmless physiological inflammation due to blastocyst implantation and tolerance of endotoxin during blastocyst implantation remains as an open question.

Systemic and intrauterine infection and inflammation have been linked to early and late pregnancy loss. In our studies, mice receiving higher dosages of LPS on day 5 showed complete resorption of implantation sites on day 8 suggesting that LPS may induce damage to the decidua either directly or indirectly. These findings agree with those of Orgando and colleagues who also found embryonic resorption on day 8 after LPS treatment (i.p.) on days 6 or 7 of pregnancy in mice [55]. Compared to the LPS group, inhibition of blastocyst implantation or resorption of implantation sites were not noted in the MPLA-treated group. This was consistent with studies by others that MPLA is virtually non-inflammatory compared to intact LPS [30,62]. Furthermore, we provide evidence that in ovariectomized as well as in pregnant mice, LPS, but not MPLA, induced the uterine production of *MyD88* and its downstream inflammatory cytokines genes such as *Tnfα*, *Il6*, and *Il1β*, but not *Trif*. These findings revealed that perhaps the inflammatory effects of LPS in the uterus and at the EISs are primarily caused by undue activation of MyD88-dependent pathway. This is consistent with previously reported findings that administration of LPS during early pregnancy increases the uterine expression of *Tnfα*, *Il6* and Il1β and has negative effects on the outcome of pregnancy [9,20].

Despite the harmful effects of LPS during pregnancy, only limited research has been conducted to develop a LPS detoxification strategy, which could be an attractive approach to protect pregnancy against endotoxin. To date, enzymatic deacylation by the AOAH enzyme [27,63] and dephosphorylation by APs [38,64] have been attributed to the loss of Lipid A toxicity. AP activity has been demonstrated in the uterus of various species where the enzyme is viewed as a maker of decidualization [32,48]. However, its biological relevance in the uterus is not understood. Using qRT-PCR, *in situ* hybridization and enzyme inhibition assay tools we demonstrated that the uterine epithelium prior to implantation and the decidua following implantation produce TNAP. Its expression in the uterine epithelium was attenuated prior to blastocyst implantation and elevated in the decidua following blastocyst implantation. This is consistent with a previous report showing the mouse uterus throughout pregnancy only expresses transcripts of *Alpl* that encodes TNAP [32]. An important variance between this study and our recent report in the hamster is that while mouse preimplantation uterine epithelium produces only the TNAP isozyme and whose expression declined during the receptive period, the hamster uterine epithelium produces two types of AP isozymes, TNAP and gIAP, and their expressions and activities showed induction during uterine receptivity [48]. Subsequent to our finding that AP is formed in uterine epithelial and decidual cells, we established that uterine TNAP is endowed with LPS dephosphorylation activity, and uterine LPS dephosphorylation sites coincide with the LPS sensing and uterine TNAP production sites. This perhaps provides one of the important explanations for considering the LE and decidua following implantation as the major sites of innate defense barriers in the uterus during pregnancy against endotoxin produced by incoming or resident bacteria [65]. Upon analyzing the contribution of uterine AP to LPS dephosphorylation, we found that treatment of pregnant animals with bIAP markedly decreased LPS-induced pregnancy complications during early pregnancy. Studies based on the levamisole sensitivity toward TNAP ascertained that circulating bIAP has reached the EIS following its systemic injection. However, direct effect of bIAP on LPS dephosphorylation could not be assessed *in vivo* since endotoxin measurement assay cannot discriminate between lipid A and monophosphoryl lipid A [31,66]. However, inorganic phosphate release from LPS by bIAP has been clearly demonstrated *in vitro* [29]. Our findings are comparable to bIAP’s preventative role in sepsis-induced mortality in mice [38] or acute kidney injury in humans [39,40], secondary peritonitis in mice [66], and necrotizing enterocolitis-associated intestinal injury [67]. Note, however, that our approach to detoxify LPS could not deal with the bacteria from which LPS is derived. Furthermore, pharmacokinetics and safety concerns of bIAP treatments in pregnant mice need to be carefully studied.

Collectively, our results provide clear evidence that the uterus possesses mechanisms for sensing LPS, as well as its detoxification by AP. However, endogenous uterine AP may not be sufficient during chronic or an inordinate levels of LPS. Intrauterine and systemic infections have been linked to the incident of early pregnancy losses (EPLs) and preterm births (PTBs) from pregnancies achieved naturally or by assisted reproductive technologies [1,68,69]. Results from clinical studies using antibiotics to reduce the rate of EPL and PTB by killing bacteria were mixed and disappointing [70,71]. Given that AP is the body’s own natural enzyme for dephosphorylation of LPS, the use of supplemental AP isozyme could be an attractive treatment option in women that are at high risk of developing pregnancy complications due to endotoxin exposure. A recent study has demonstrated that AP dephosphorylates not only LPS, but also bacterial flagellin and CpG DNA [72], highlighting a potential clinical utility of AP isozyme in detoxifying other bacterial-derived toxin molecules.
